# Residues in Transmembrane Segments of the P2X4 Receptor Contribute to Channel Function and Ethanol Sensitivity

**DOI:** 10.3390/ijms21072471

**Published:** 2020-04-02

**Authors:** Maya Popova, Larry Rodriguez, James R. Trudell, Sylvia Nguyen, Michael Bloomfield, Daryl L. Davies, Liana Asatryan

**Affiliations:** 1 Department of Pharmacology and Pharmaceutical Sciences, University of Southern California School of Pharmacy, 1985 Zonal Avenue, Los Angeles, CA 90089, USA; mayapopova37@gmail.com (M.P.); larryrod@usc.edu (L.R.); sylvia6189@gmail.com (S.N.); mbloomfield77@gmail.com (M.B.); 2 Department of Anesthesia, Beckman Program for Molecular and Genetic Medicine, Stanford University, Stanford University Medical Center, Stanford, CA 94305, USA; trudell@stanford.edu; 3 Titus Family Department of Clinical Pharmacy, University of Southern California School of Pharmacy, 1985 Zonal Avenue, Los Angeles, CA 90089, USA; ddavies@usc.edu

**Keywords:** purinergic (P2X4) receptor, TM1 and TM2 segments, ethanol and agonist action, mutagenesis, molecular model

## Abstract

Mouse models of alcohol use disorder (AUD) revealed purinergic P2X4 receptors (P2X4Rs) as a promising target for AUD drug development. We have previously demonstrated that residues at the transmembrane (TM)–ectodomain interface and within the TM1 segment contribute to the formation of an ethanol action pocket in P2X4Rs. In the present study, we tested the hypothesis that there are more residues in TM1 and TM2 segments that are important for the ethanol sensitivity of P2X4Rs. Using site-directed mutagenesis and two electrode voltage-clamp electrophysiology in *Xenopus* oocytes, we found that arginine at position 33 (R33) in the TM1 segment plays a role in the ethanol sensitivity of P2X4Rs. Molecular models in both closed and open states provided evidence for interactions between R33 and aspartic acid at position 354 (D354) of the neighboring TM2 segment. The loss of ethanol sensitivity in mixtures of wild-type (WT) and reciprocal single mutants, R33D:WT and D354R:WT, versus the WT-like response in R33D-D354R:WT double mutant provided further support for this interaction. Additional findings indicated that valine at TM1 position 49 plays a role in P2X4R function by providing flexibility/stability during channel opening. Collectively, these findings identified new activity sites and suggest the importance of TM1-TM2 interaction for the function and ethanol sensitivity of P2X4Rs.

## 1. Introduction

Alcohol abuse and alcoholism (alcohol use disorder: AUD) continues to present a serious health and economic burden worldwide [1,2], due in part to the lack of effective medications for treatment and/or prevention. However, medication development is complicated by a lack of understanding of the targets, target sites, and mechanisms of ethanol action (i.e., alcohol intoxication) in the central nervous system (CNS). Growing evidence suggests that ethanol affects purinergic P2X4 receptors (P2X4Rs), a member of the P2XR superfamily (for review, see [3]). Of the seven family members, P2X4Rs are the most abundant P2XR subtype in the CNS [4,5]. P2X4Rs have been found in reward circuitry, specifically the mesolimbic and mesocortical pathways [6]. Gene expression profiling studies have suggested that P2X4R expression may be involved in innate alcohol preference, and, indeed, reduced regional levels of *p2rx4* mRNA were found in alcohol-preferring vs. alcohol non-preferring rats [7] as well as high alcohol drinking vs. low alcohol drinking rats [8].

In agreement with these findings, studies from our group revealed that ethanol intake was higher in P2X4-knockout male mice compared to their wild-type (WT) counterparts, in both intermittent limited access and 24-hr access drinking models [9]. Additionally, within the P2XR family, P2X4Rs are the most ethanol-sensitive subtype; in vitro, P2X4Rs are inhibited by behaviorally relevant concentrations of ethanol (e.g., 17 mM which is equal to 0.08% blood ethanol, or the legal limit to drive in the United States of America) [10,11,12,13]. Together, these findings indicate that P2X4Rs and ethanol are related in the behavioral, cellular, and molecular level, and therefore represent an important therapeutic target for pharmacological strategies aimed at the prevention and/or treatment of AUD.

Functional P2XRs are homomeric (formed from a single P2X subtype) or heteromeric (formed from different P2X subtypes) trimers. Structurally, each subunit consists of two intracellular N- and C-termini, two transmembrane (TM) segments, and a large extracellular domain (ectodomain) [14]. Interactions between the TM1 and TM2 segments have been shown to play a major role in the function of P2X4Rs. Tryptophan and cysteine scanning studies of both TM1 and TM2 revealed that the upper regions of each segment undergo substantial rearrangement and adopt α-helical secondary structures during channel gating [15,16,17]. Furthermore, interactions between the TM1 region of one subunit and the TM2 region of another subunit have been shown to regulate the gating and ion conductance of P2X4Rs, specifically the lower ends of the TM segments [18]. Studies have suggested that homomeric P2X4Rs contain a putative ethanol activity site formed by residues located at the ectodomain–TM interfaces [19], aspartic acid at position 331, and methionine at 336 located in the upper part of the TM2 segment [12], and two tryptophan residues at position 50 at TM1–ectodomain interface and position 46 in the TM1 segment [12]. In this regard, homology models of the rat P2X4R [12,20] built using open and closed conformations of zebrafish P2X4R as templates [21,22] suggest that residues located in the TM1 segment influence ethanol interactions with P2X4Rs [12] and resolved potential interactions between residues at the TM2–ectodomain interface. These findings provide a strong support for the notion that there are multiple sites in the TM1 and TM2 segments of P2X4Rs responsible for receptor function and ethanol sensitivity. Despite these advances, we still lack a clear understanding of how other residues in the TM1 and TM2 segments function and/or interact during ethanol action in the P2X4R.

In this study, we employed an alanine scanning strategy on the TM1 segment of the P2X4R to identify novel residues responsible for ethanol sensitivity and receptor function. We explored possible interactions between the residues at the lower portions of TM1 and TM2 segments with additional site-directed mutagenesis and molecular modeling. To further understand the role of these residues in receptor function and ethanol response, we also tested the effects of ivermectin (IVM) on several mutant receptors based on our previous studies that found partially overlapping sites for ethanol and IVM, and its recognition for interacting favorably with the open P2X4R conformation [19,23].

## 2. Results

### 2.1. TM1 Alanine Scan Revealed Residues That Are Important for Receptor Function

**Figure A1:**
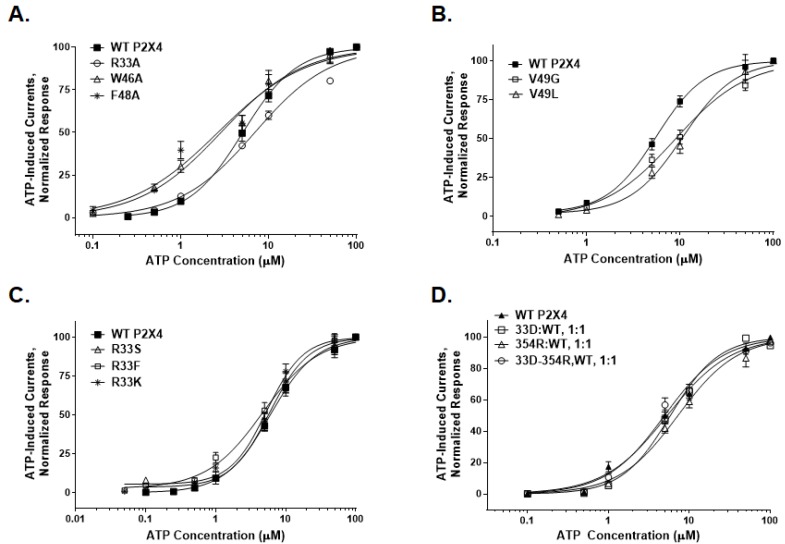
ATP concentration-response curves for WT and select P2X4R mutants. (**A**) Hill slopes were significantly reduced for F48A, W46A and R33A mutants (See Table 1). There was a left-shift for F48A and W46A mutants in the ATP concentration-response curves and a tendency for right-shift for R33A compared to that of the WT receptor. (**B**) A right-shift in the ATP concentration-response curves shown for V49G and V49L mutants. (**C**) Position 33 mutants demonstrated WT-like ATP concentration responses. (**D**) WT-like ATP concentration responses for position 33 and 354 reciprocal mutant:WT receptor mixtures.

Amino acid residues within the TM1 segment of rat P2X4Rs, positions 29 through 49, were individually mutated to alanine (A), excluding alanine residues at positions 34 and 41, which were mutated to tryptophan (W). All mutant receptors were tested for changes in agonist response (Table 1). Apart from valine (V) 49 to alanine (V49A), all mutants produced measurable ATP-gated currents. One-way ANOVA found a significant effect of mutations on the maximum inducible currents (Imax). Individual *t*-tests indicated significant differences in Imax values between the WT P2X4R and number of mutants (Table 1). Further analysis found significant changes in EC_50_ values for F48A, W46A, and L37A mutants and significantly lower values for the Hill slopes for F48A, W46A, G45A, and R33A mutants when compared to that of the WT P2X4R (Table 1, see also Appendix A Figure A1A for some of the mutants).

**Figure 1:**
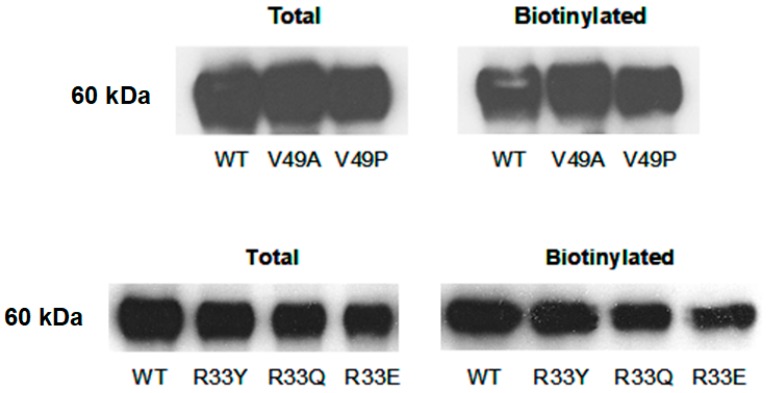
Mutations at positions 49 and 33 do not significantly affect total and surface expression of mutant P2X4Rs. Representative Western Blots showing the P2X4R bands at ~60 kDA for total and biotinylated fractions for WT and mutant receptors.

Based on the Imax, EC_50,_ and Hill slope values, more notable changes occurred in V49A, F48A, and W46A mutants. We have discussed changes and the role of W46 residue in P2X4R function in our previous publication [21]. Not undermining the role of residue at position 48, which can be studied in more detail in the future, in this study we focused on V49 as mutation of this residue to alanine resulted in a loss of function. To determine if this effect in V49A mutant was due to a lack of receptor expression, we performed surface biotinylation and subsequent Western Blot analysis. We found that the surface expression of this mutant receptor was similar to that of the WT receptor (Figure 1). We next tested whether position 49 was involved in the function of a putative hinge region theorized to play a role in the stability of the alpha-helical structure of the TM1 segment [24]. This was accomplished by mutating the valine at position 49 to proline (P) or glycine (G), as proline and glycine are known to be alpha-helix destabilizing residues [24,25]. In addition, leucine (L), an alpha-helix stabilizing residue [24], was substituted for valine (V49L). 

ATP elicited a negligible response in V49P but measurable currents in V49G and V49L mutants (Table 2). There was a right-shift in ATP-concentration response curves for V49G and V49L mutants with significantly higher EC_50_ values (Table 2; Appendix A Figure A1B). Like the V49A mutant, the V49P mutant was readily expressed on the surface of the oocytes (Figure 1), suggesting that trafficking of the mutant receptor to the surface was not affected by mutagenesis.

### 2.2. Alanine Scan of the TM1 Revealed Sites for Ethanol Action

**Figure 2:**
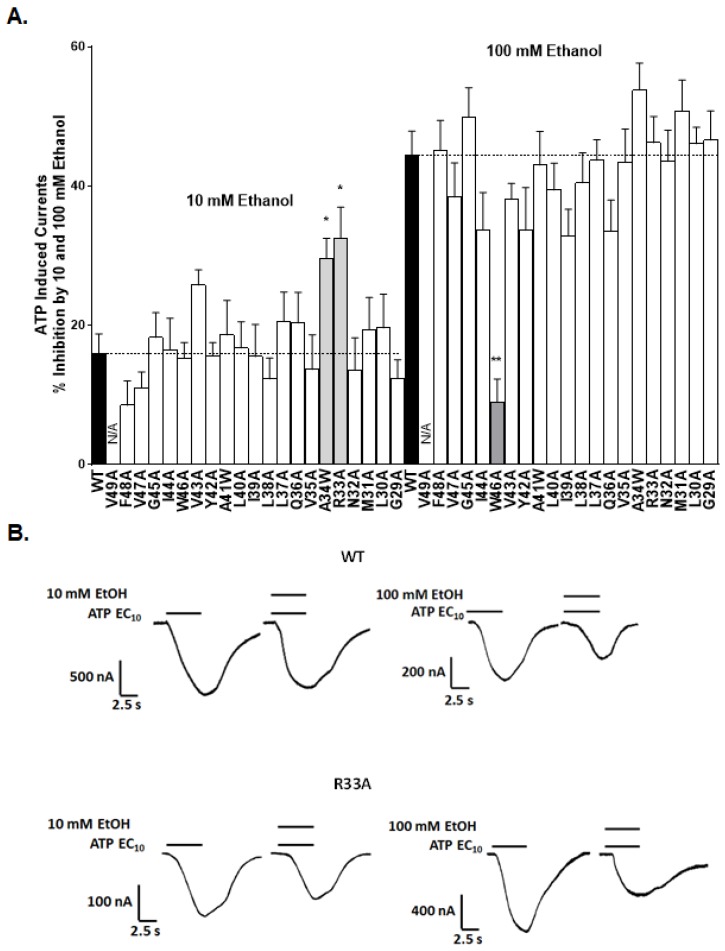
Effects of 10 mM and 100 mM ethanol in WT and TM1 segment mutant P2X4Rs. (**A**) A bar graph of ethanol responses. The response to 10 mM ethanol was significantly increased in the R33A and A34W mutants’ receptors, and the inhibitory effect to 100 mM ethanol was significant decreased in the W46A mutant receptor. The data are presented as mean ± SEM, *n* = 5–18. * *p* < 0.05 and ** *p* < 0.001 compared to ethanol effects in the WT P2X4R. (**B**) Representative ATP-induced current tracings for the WT and R33A mutant P2X4Rs; the effects of 10 and 100 mM ethanol are shown.

Continuing our alanine scanning mutagenesis strategy, we next investigated how mutations in the TM1 region affected the inhibitory effects of ethanol on P2X4R function. We focused on two concentrations of ethanol (10 mM—low, behaviorally relevant; 100 mM—high, pharmacologically challenging). In good agreement with the previously reported values [12,20], ethanol significantly inhibited EC_10_ ATP-activated currents in WT P2X4Rs (Figure 2A,B). The degree of ethanol inhibition in WT receptors was 16 ± 3% for 10 mM ethanol and 44 ± 4% for 100 mM ethanol.

In the tested mutants, we found that the inhibitory effect of ethanol at 10 mM was significantly increased compared to the WT receptor in R33A and A34W mutants (Figure 2A). However, the inhibitory effects of ethanol at 100 mM were similar in WT and most of the mutant receptors, including R33A and A43W (Figure 2A), with the exception of the W46A mutant. Substitution of tryptophan at 46 with alanine significantly reduced the inhibitory response to 100 mM ethanol (Figure 2A). We have previously studied the role of position 46 in ethanol action [20]. For this study, we chose to focus on arginine at position 33 because of its positive charge, and its potential for interactions with other residues.

### 2.3. Physical-Chemical Properties of Residues at Position 33 Determine Receptor Function and Ethanol Sensitivity

The WT arginine residue at position 33 was mutated to amino acids with different physical-chemical properties in order to examine whether the polarity and/or size of the individual side-chains play a role in the (1) agonist properties and/or the (2) ethanol sensitivity of the receptor.

#### 2.3.1. Receptor Function

Mutating the positively charged bulky residue arginine at position 33 to another positively charged residue, lysine (K), produced a mutant receptor with I_max_ currents and an EC_50_ value similar to WT P2X4Rs (Table 2). Substitutions with small polar serine or non-polar residues valine, cysteine (C), leucine (L), or phenylalanine (F) also produced mutants with WT-like agonist responses (Table 2, Appendix A Figure A1C). Mutating the positively charged arginine at position 33 to the negatively charged glutamic acid (E) or non-charged, bulky polar residues, such as glutamine (Q) or tyrosine (Y), yielded very small I_max_ currents compared to WT, precluding the determination of their EC_50_. The total and surface expression of these mutants were not different compared to the WT P2X4R (Figure 1), suggesting that the charge, rather than the polarity or size, of the residue at position 33 is important for the normal receptor function.

#### 2.3.2. Ethanol Response

**Figure 3:**
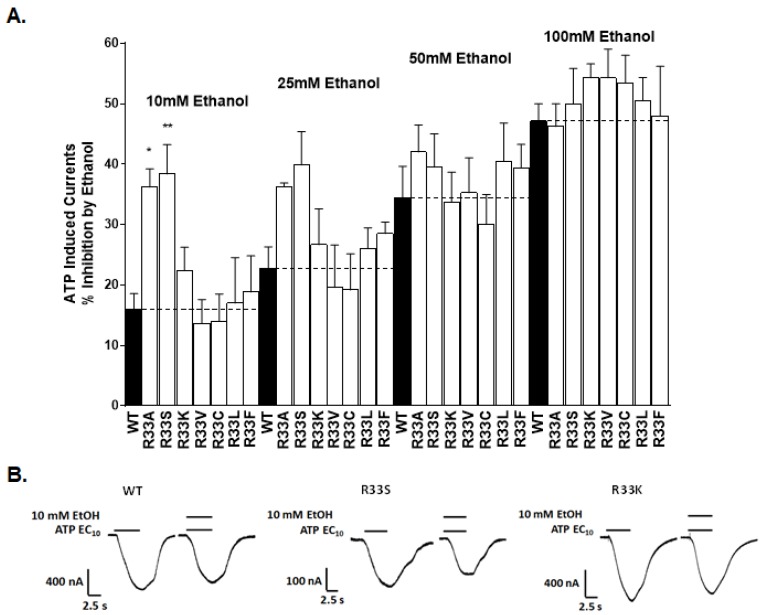
Effects of ethanol (10–100 mM) in mutant receptors at position 33. (**A**) A bar graph of ethanol responses. Alanine or serine substitution at position 33 increased the inhibitory effect of ethanol at low concentrations (10 mM and 25 mM). The increases were significant for 10 mM but not 25 mM ethanol. The data are expressed as mean ± SEM, *n* = 4–17. * *p* < 0.05, ** *p* < 0.005 compared to ethanol effects in the WT P2X4R. (**B**) Representative ATP-induced current tracings for the WT, R33S, and R33K mutant P2X4Rs; the effects of 10 mM ethanol are shown.

We tested the effects of increasing ethanol concentrations (10, 25, 50, and 100 mM) of ethanol in position 33 mutants (Figure 3). As reported above, alanine substitution at position 33 (R33A) produced a mutant receptor that had a significantly greater degree of inhibition at low 10 mM ethanol concentration compared to WT P2X4Rs. Consistently, substituting serine (S), another small, polar amino acid, for large arginine (R33S) resulted in a similar increase in the inhibitory response to 10 mM ethanol compared to the WT (Figure 3). Further increases in ethanol inhibition in R33A and R33S mutants were also observed at 25 mM ethanol; however, these effects were not significant. No differences between the effects of these two mutants and WT receptors were observed at 50 and 100 mM ethanol. The substitution of non-polar amino acids valine (R33V), cysteine (R33C), leucine (R33L), and phenylalanine (R33F), for the large, positively charged arginine at position 33 produced mutant receptors with WT-like ethanol sensitivities at all tested ethanol concentrations (Figure 3). Finally, substituting the positively charged lysine (K), a residue with similar electrostatic properties as WT arginine (R33K), also resulted in WT-like ethanol responses. The molecular weight of the residue at position 33 negatively correlated with the ethanol effect (Pearson’s r = −0.47 and −0.54 for 10 and 25 mM ethanol, respectively). These data suggested that the size is the main property at position 33 that determines the ethanol sensitivity of P2X4Rs.

### 2.4. Residues That Are Important for Agonist Sensitivity and Ethanol Effects Identified in Homology Models

**Figure 4:**
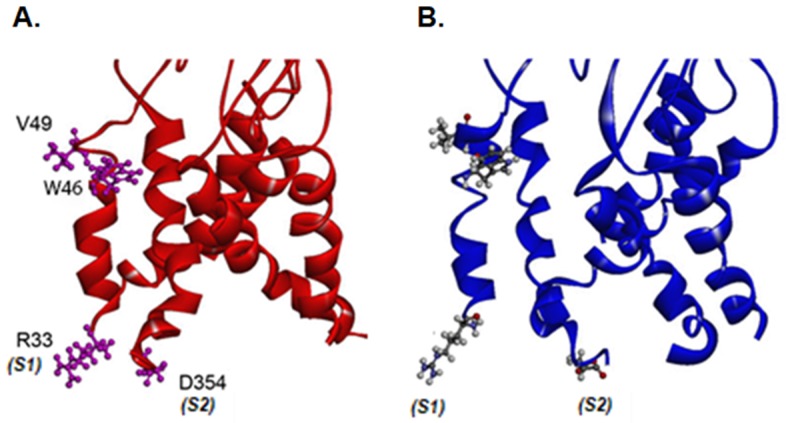
Homology models of rat P2X4Rs based on the closed (4DW0) and open (4DW1) zebrafish structures [22]. (**A**) A model illustrating the transmembrane (TM) segments in the closed state (red solid ribbon of backbone with residues at positions 33, 46, and 49 in the TM1 segment of one subunit as well as 354 in the TM2 segment of the neighboring subunit depicted as a ball and stick. Residue 33 of subunit 1 (S1) and 354 of subunit 2 (S2) face each other on the closed structure. (**B**) A model illustrating the TM segments in the open state (blue line ribbon of backbone) with the same residues shown in A. The residues at positions 33 (S1) and 354 (S2) face away from each other.

We used homology models of the rat P2X4Rs, based on the structures of the zebrafish P2X4R [22], as a template to visualize the potential interactions of TM1 segment V49 and R33 in both closed and open configurations (Figure 4A,B). As hypothesized, the closed conformation of the P2X4R shows that V49 is located in a hinge region of an alpha-helical structure, within close proximity to W46 of the same TM1 segment (Figure 4A). In the open conformation, a shift appears to occur, which results in these two residues facing one another (Figure 4B). The model also illustrates that, in the closed conformation, positively charged R33 in the TM1 segment of one P2X4 subunit and negatively charged residue D354 at the end of the TM2 of the neighboring subunit are in close proximity (estimated distance at 8 Å), potentially forming a salt bridge (Figure 4A). The interaction between R33 and D354 seems to be conserved but is weaker in the open conformation, where both residues are farther apart (10 Å), as illustrated in Figure 4B. We theorized that this putative salt bridge may be stabilizing the lower end of each subunit during the closed state and that this interaction is somewhat weakened during the transition from the closed to the open state, allowing D354 to alter the flow of positively charged ions.

### 2.5. Arginine 33 and Aspartic Acid 354 Interactions

**Figure 5:**
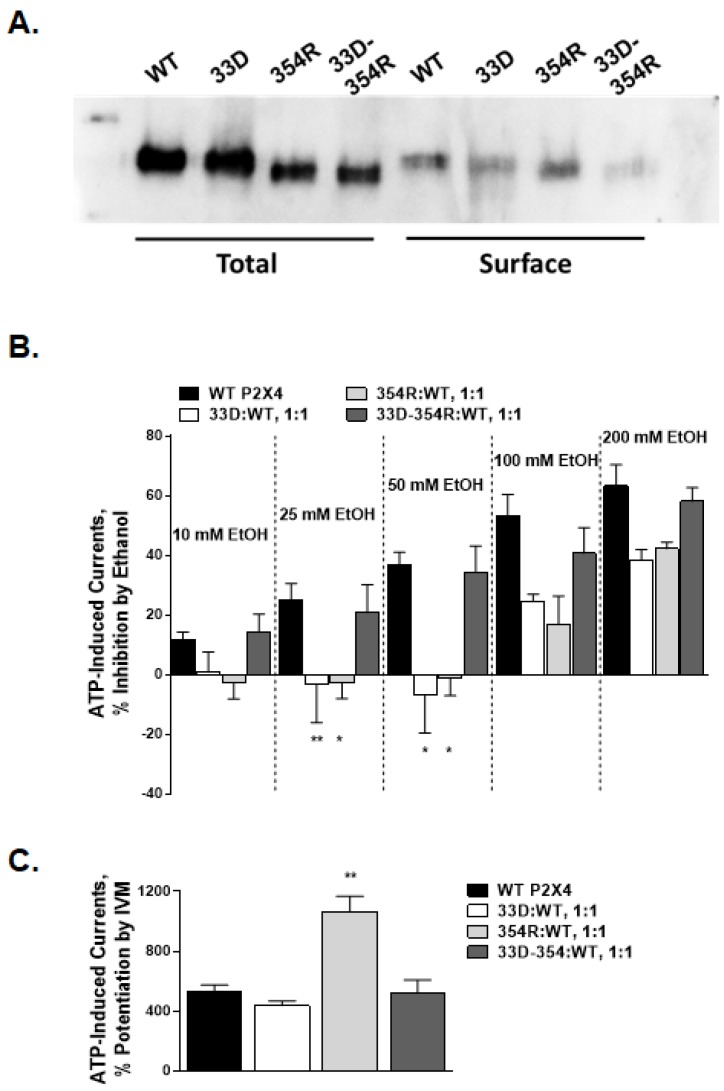
Effects of reciprocal mutations at position 33 and/or 354 as well as the mixtures of reciprocal and WT on ethanol and ivermectin (IVM) responses. (**A**) A representative Western Blot showing the total and surface expression (~60 kDA) for R33D, D354R, and R33D-D354R mutant receptors. The expression of the mutants was not much different compared to the WT receptor. (**B**) A bar graph of the responses to 10–200 mM ethanol for mutant:WT receptor mixtures (at a ratio of 1:1). The data are expressed as mean ± SEM; * *p* < 0.05, ** *p* < 0.01 compared to ethanol effects in the WT P2X4R. (**C**) A bar graph of the responses to 3 µM IVM for mutant:WT receptor mixtures. The data are expressed as mean ± SEM; * *p* < 0.05, ** *p* < 0.01 compared to IVM effects in the WT P2X4R.

To test the potential interactions between R33 and D354, we reciprocally mutated the R33 and D354 residues to their putative interaction partner, generating single mutants, R33D, and D354R, as well as the double reciprocal mutant R33D-D354R. In all mutants, ATP evoked negligible currents, and while the surface expression of these mutants was lower, mutant receptors were expressed in oocytes (Table 3; Figure 5A), suggesting that these mutations hindered receptor activity. To overcome these functional issues, we adopted a strategy from Silberberg et al. [15] where WT and non-functional mutant cRNAs are mixed at an equal ratio and injected into oocytes.

While this mixing strategy resulted in substantially smaller Imax values, we saw no significant changes in EC_50_ and/or Hill slopes from those of the WT P2X4R (Table 3, Appendix A Figure A1D). Surprisingly, the inhibitory effects of ethanol were profoundly different in these mutant receptors (Figure 5B). When compared to the WT responses, ethanol inhibition in the range of 10–50 mM was abolished in 354R:WT and 33D:WT receptor mixes (Figure 5B). One-way ANOVA showed a significant effect of the mutations on ethanol inhibition across all ethanol concentrations, with significant individual differences between mutant (354R:WT and 33D:WT mixes) and WT receptors at 25 and 50 mM ethanol. Remarkably, the double reciprocal mutant and WT receptor mix, i.e., 33D-354R:WT, consistently showed ethanol inhibition similar to that of the WT receptor. These data suggest that the 33D and 354R mutant receptors heteromerized with the WT P2X4R to form functional channels, and that these mutations likely affect ethanol sensitivity.

To further characterize the interaction between R33 and D354, we also tested the effects of IVM on mutant-WT receptor mixtures. Studies have shown that IVM potentiates the WT P2X4Rs by interacting favorably with the open receptor conformation. If R33 and/or D354 were involved in an interaction that affects the transition between the closed and open states, the effects of IVM would be significantly augmented for one or both mutants. The potentiating effect of 3 µM IVM in the 33D:WT mixture was similar to the effect seen for the WT, while the ATP-evoked response was potentiated approximately 5-fold in 354R:WT mixture (Figure 5C). In contrast, the double reciprocal 33D-354R:WT mixture responded to IVM similar to the WT receptor (Figure 5C). These results suggest that D354 plays a role in regulating ion conductance.

## 3. Discussion

We previously identified residues at the ectodomain–transmembrane (TM) interface (aspartic acid at position 331 and methionine at 336) and the TM1 segment (tryptophan at position 46) of P2X4Rs that play a role in the ethanol sensitivity of the receptor [12,20]. The present study revealed new residues in the TM1 segment of P2X4Rs that play a role in the receptor function and resolved a potential interaction between the TM1 and TM2 segments that affects ethanol sensitivity. Among these new residues, stronger and more consistent changes in Imax, EC_50_, and/or Hill slope values were found for V49, F48, and W46 residues. We further focused on V49 responses to ATP.

### 3.1. Function of Valine at Position 49 in the upper Portion of the TM1 Segment

The alanine scan of TM1 and follow-up mutational studies provide insight into the role of valine at position 49 in receptor function in P2X4Rs. Alanine substitution at this position caused a loss of function despite no change in surface expression of the mutant receptor. These findings are in agreement with previous studies, which showed that mutating the valine residue at position 49 to either alanine or cysteine affected the response to agonist [16].

Previous studies, using circular dichroism and nuclear magnetic resonance spectroscopy, found that valine, a β-branched residue found in the TM segments of membrane proteins, may contribute to receptor function by providing conformational flexibility and/or helix destabilization at various stages of the protein activation cycle [24]. Thus, it is possible that in the P2X4R, valine at position 49 provides the upper portion of the TM1 segment with the flexibility needed for reorganization during the opening or closing of the channel. Studies on other proteins found that substituting an alanine for valine caused tighter protein–protein packing and increased α-helical stability due to the reduction in side-chain volume and strain [26]. The changes in flexibility and packing could reflect, in part, changes in the orientation and interaction of the neighboring aromatic residues produced by substituting alanine for valine. In addition, previous studies found that the aromatic residues in the upper half of the TM1 segment near position 49, which includes tryptophan 46 and tryptophan 50, play an important role in the three-dimensional organization of P2X4Rs [27]. Therefore, position 49 could alter the orientation of these nearby aromatic residues and thus affect the channel function. A shift in the position of V49, noted in the molecular model of the open conformation of the P2X4R, further supports this hypothesis.

To test whether position 49 was in fact important for the stability of the alpha-helical structure of the TM1 segment, we mutated the valine to known alpha-helix destabilizing residues (proline or glycine) and or to an alpha-helix stabilizing residue (leucine) [24,25]. Substitutions to alpha-helix destabilizing residues either reduced (glycine) or eliminated (proline) the agonist response. The presence of an alpha-stabilizing leucine also resulted in decreased agonist response. Previous studies have shown that the substitution of V49 with the hydrophobic residue tryptophan had no effect on ATP-induced currents or IVM potentiation when compared to the WT receptor [23], which suggests that the effects we see in V49 are not directly related to P2X4R gating. Valine, most probably, provides a certain level of stability to the alpha helix of the TM1 segment, and this may be due to its interactions with neighboring residues, in part from the TM1 segment, such as F48, V47, and W46. It is likely that a higher or lower level of stability respectively introduced by the mutations to glycine or leucine may have similarly affected the receptor function towards lower activity, as reflected in the EC_50_/Imax values of the corresponding mutants. Collectively, these findings suggest that position 49 provides flexibility/stability to the upper portion alpha-helix of the TM1 segment, which is required for the proper function of the P2X4 channel.

### 3.2. Arginine at Position 33 in the TM1 Segment in Ethanol Action

Prior studies suggested that there are multiple sites of ethanol action in P2X4Rs. We identified D331 and M336 in the ectodomain–TM2 interface and W46 in the TM1 segment as sites of ethanol action and/or modulation in P2X4Rs [12,20]. Mutations at positions 331 and 336 decreased the sensitivity of P2X4Rs to a broad range of ethanol concentrations extending from the behaviorally relevant or intoxicating (10–50 mM) to potentially toxic concentrations (100–200 mM). In contrast, mutations at position 46 only affected the sensitivity of the receptor to high ethanol concentrations (>50mM). Consistent with previous findings, the results of the current study suggest that there are additional sites with different sensitivities to ethanol in the lower region of the TM1 segment of P2X4Rs, i.e., positions 33 and 34. Increased sensitivity to 10 mM ethanol suggests that these residues may be important for the effects of the lower, behaviorally relevant ethanol concentrations.

Initial alanine substitution studies affected ethanol responses at two low but behaviorally relevant concentrations, 10 and 25 mM. Therefore, in the present study, we performed an in-depth investigation of the physical-chemical requirements of position 33 for the ethanol sensitivity of P2X4Rs. We found an inverse relationship between the molecular size of the amino acid residue at position 33 and ethanol sensitivity. This response was independent of the polarity of the residue. Despite the fact that we could not assess the ethanol sensitivities of all tested residues due to the minimal Imax currents of several of the mutants (i.e., glutamic acid, glutamine, and tyrosine), these findings support the conclusion that the size of the residue at position 33 is a key factor influencing the ethanol sensitivity of P2X4R. Our previous findings also suggested a key role for the molecular size, and not polarity, of the residue at position 46, with the difference that there was a positive correlation with ethanol inhibition [20]. In contrast, polarity, not molecular size, at positions 331 and 336 in the ectodomain–TM2 interface of P2X4Rs plays a role in ethanol sensitivity [12]. Together, these findings suggest that different physical-chemical properties influence the ethanol sensitivity of P2X4Rs and that these differences may be region specific. Similar regional differences in the effects of physical-chemical properties on ethanol sensitivity have been reported in glycine receptors [28,29,30,31], which suggests that this is a general phenomenon that extends across ligand-gated ion channel super-families.

### 3.3. Interaction between TM1 Arginine at Position 33 and TM2 Aspartic Acid at Position 354 in Channel Functioning

Studies have shown that the TM1 segment of the P2X4R has a limited contribution to ion conduction by itself [32,33,34]. However, prior work has also shown a conserved mechanism within the P2XR family where the TM segments of the receptor subunits rearrange during channel opening or closing, allowing for residues from different subunits to interact with one another [21,22,23,33,34,35]. We hypothesized that the positively charged arginine at position 33 of the TM1 segment may interact with a negatively charged aspartic acid of the TM2 segment of a neighboring subunit. To visualize this potential interaction, we built homology models with the zebrafish P2X4R serving as a template. Both the closed and open structures of the P2X4Rs highlight the close proximity of arginine (R) at position 33 in the TM1 segment of one P2X4 subunit and aspartic acid (D) at position 354 in the TM2 subunit of another subunit (Figure 4A), supporting the idea of a potential interaction between the two residues. We tested this finding by making reciprocal interaction mutations, i.e., R33D and D354R mutants, however those turned to be non-functional. We then adopted a strategy previously used by Silberberg et al. [15] which produced responses from non-functional mutations via the incorporation/mixing of WT receptor cRNA, mixed in equal ratios. These functional mutant receptor mixtures, R33D:WT and D354R:WT, exhibited WT-like agonist properties (as observed by EC50 and Hill slope values), although the changes seen in the ethanol sensitivity of these receptor mixtures indicate successful incorporation of mutant subunits in these functional receptors. The inhibitory effects of sub-100 mM ethanol were abolished in the single reciprocal mutations, suggesting that the interaction between the lower portions of the TM segments plays a role in the ethanol sensitivity of P2X4Rs. More importantly, the double mutant R33D-D354R:WT mixture demonstrated WT-like responses to ethanol over the whole range of concentrations. Interestingly, the physical site for ethanol activity did not seem to be disturbed by a double-reciprocal mutation of these residues.

We have previously found that sites for ethanol and IVM partially overlap, i.e., that IVM antagonizes the inhibitory action of ethanol [19]. Therefore, we tested whether IVM potentiation would be disturbed in these reciprocal mutant mixtures. Consistent with the response to ethanol, there was no change in IVM potentiation in the double reciprocal mutant or the single R33D:WT mutant, which retained WT-like responses to IVM. Only the reciprocal mutation of position 354 significantly increased IVM potentiation (seen in D354R:WT receptor mixtures). These results are consistent with previous studies where the tryptophan scanning mutation of R33 did not affect IVM potentiation and the tryptophan substitution of D354 was non-functional [15]. It is unlikely that mutating the TM2 segment residue (D354) creates a conformational change affecting IVM binding pocket, as the double reciprocal mutation retains WT-like IVM responses, suggesting a WT-like interaction between the TM1 and TM2 segment during channel opening.

These findings provide new insights into the importance of interactions between the lower portions of the TM1 and TM2 segments in P2X4Rs for ethanol action. In combination with the findings from our molecular models, these results suggest that the interactions between R33 and D354 residues affect the transition from closed to open conformation during channel opening. The presence of an aspartic acid residue in TM2 is conserved among all P2X subtypes [21] and has been shown to play a significant role in ion conductance [36]. Moreover, recent studies suggest that the lower part of the TM2 segment changes shape during activation [37]. The systemic probing of the TM2 domain using the substituted cysteine accessibility method (SCAM) presented evidence that the TM2 domain was the primary pore-forming part of P2XRs, which is in agreement with the crystal structure [21,22]. Using combination of SCAM with a thiol-reactive probe Cd^2+^, Kracun et al. identified D349 in P2X2Rs that provides movement to the pore-lining regions of the TM2 during channel opening [37]. Interestingly, P2X2R residue D349 corresponds to D354 in P2X4R [37]. As such, it is plausible that during channel opening, the interaction between R33 and D354 is weakened, thus allowing D354 to alter the flow of positively charged ions. With these considerations in mind, it is logical to conclude that non-polar substitutions at position 33 would not change the nature of the interaction with the residue at position 354 and therefore, these mutants would behave like WT in terms of ion conductance (Imax). In the case of substitutions of position 33 with negatively charged or polar residues, an interaction with D354 is altered, causing a loss of receptor function. Collectively, these results suggest that an interaction between TM1 and TM2 segments of P2X4Rs occurs in the closed state, which is altered during channel opening, with D354 driving the conformational change and ion conductance and position 33 contributing to the channel stability.

## 4. Materials and Methods

### 4.1. Materials

Adenosine 5′-triphosphate disodium salt, ethanol (190 proof, USP), and other chemicals were purchased from Sigma Co. (St. Louis, MO, USA). All other chemicals were of reagent grade.

### 4.2. Isolation of Xenopus Laevis Oocytes and cRNA Injections

*Xenopus* oocytes were isolated from *Xenopus Laevis* (Nasco, CA, USA) and maintained as described previously [20]. Stage V and VI oocytes were selected, rinsed and stored in incubation medium containing (in mM), NaCl 96, KCl 2, MgCl_2_ 1, CaCl_2_ 1, HEPES 5, theophylline 0.6, pyruvic acid 2.5, with 1% horse serum and 0.05 mg/mL gentamycin. The following day, oocytes were injected (32 nl, 20 ng/oocyte) into the cytosol with cRNA encoding rat WT or mutant P2X4R or the mixtures of WT and non-functional mutants (GenBank accession no. X87763). The injections were performed with Nanoject III Nanoliter injection system (Drummond Scientific, Broomall, PA, USA). The injected oocytes were stored in incubation medium at 17 °C and used in electrophysiological recordings for 1–4 days after cRNA injections.

### 4.3. Site-Directed Mutagenesis and cRNA Synthesis

The cDNA of rat P2X4R (GenBank accession no. X87763) was subcloned into pcDNA3 vector (Invitrogen, Carlsbad, CA, USA). The mutations were generated by using QuikChange II XL Site-Directed Mutagenesis Kit (Stratagene, La Jolla, CA, USA). The primers were purchased from Integrated DNA Technologies (Coralville, Iowa). Once the mutations were sequence verified (Genewiz, La Jolla, CA, USA), plasmid DNA was linearized by restriction digestion with XhoI and cRNA was synthesized using mMESSAGE mMACHINE T7 Kit (Applied Biosystems, Foster City, CA, USA) and stored at −70 °C until injection.

### 4.4. Whole-Cell Voltage Clamp Recordings

To increase the number of mutant receptors that can be efficiently screened, we used an automated computer-controlled 8-channel two electrode voltage-clamp system, *OpusXpress* (Molecular Devices, Union City, CA, USA). Oocytes were placed in 8 recording chambers (volume 200 µL), superfused with P2X buffer solution (in mM) (NaCl 110, KCl 2.5, BaCl2 1.8, HEPES 10, pH 7.5) at a rate of 3–4 mL⁄min, and impaled with 2 glass electrodes filled with 3 M KCl (0.5 to 2 MΩ). The membrane potential was held at −70 mV, and the currents were sampled at 5 KHz and filtered at 1 KHz. The currents acquired were stored on the computer for off-line analysis using Axon pClamp 9 software (Axon Instruments, Union City, CA, USA).

### 4.5. Experimental Procedures

All experiments were performed at room temperature (20–23 °C). To generate ATP-concentration curves, oocytes were exposed to 0.05–100 µM ATP range for 5 sec followed by 5–15 min washout. The effect of ethanol on P2X4R function is more robust and reliable when tested in the presence of sub-maximal concentrations of ATP (typically EC_5–20_) [19,20]. Therefore, ethanol was co-applied with EC_10_ ATP. ATP EC_10_-activated currents were measured before and after each ethanol application to take into account possible shifts in the baseline current values. Ethanol did not affect the resting membrane currents in oocytes expressing P2X4Rs in the absence of agonists or in un-injected oocytes. IVM was pre-applied to the oocytes for 1 min and then co-applied with EC_10_ of ATP.

### 4.6. Cell Surface Biotinylation and Immunoblotting

The study was performed according to procedures established in our laboratory (Perkins et al., 2009). Briefly, 2–5 days after injections with WT or mutant P2X4R cRNAs, oocytes (15 oocytes/group) were incubated with 1.5 mg/mL membrane-impermeable sulfosuccinimidyl 2-(biotinamido)-ethyl-1,3-dithiopropionate (Thermo Fisher Scientific Inc., Rockford, IL, USA) for 30 min at room temperature. Oocytes were homogenized in 500 μL of lysis buffer (40 mM Tris (pH 7.5), 110 mM NaCl, 4 mM EDTA, 0.08% Triton X-100, and 1% protease inhibitor cocktail (Sigma, St. Louis, MO, USA), and after the cellular debris and yolk were removed by centrifugation at 10,000× *g* for 10 min, 50 μL aliquots were stored at −20 °C to assess total receptor fraction. Biotinylated proteins were captured by overnight incubation with streptavidin beads (Thermo Fisher Scientific Inc., Rockford, IL, USA) at 4 °C and eluted by heating at 95 °C for 10 min in SDS loading buffer. The surface (biotinylated) and total proteins were then separated on SDS-PAGE and transferred to PVDF membranes. The membranes were then incubated overnight with polyclonal antibodies against P2X4Rs (1:2000 dilution; Alomone Labs, Jerusalem, Israel), followed by incubation with HRP-conjugated secondary antibodies. Protein bands were visualized using enhanced chemiluminescence (Thermo Fisher Scientific Inc., Rockford, IL, USA).

### 4.7. Homology Modeling

We built two new homology models of rat P2X4based on the closed (PDB ID 4DW0) and open (PDB ID 4DW1) zebrafish structures [22]. We used the ‘modeler’ module of Discovery Studio 3.5 (DS 3.5; Biovia, San Diego, CA, USA) to make 50 models of both the open and closed form, essentially as we previously described [12,20]. We chose the ‘best’ models based on force field energy. All side chain rotamers of these selected models were optimized with the ‘side chain refinement’ module of DS 3.5 while the backbone atoms were fixed. Then harmonic backbone restraints of 10 kcal/(mol x Angstrom) were applied and the models were optimized using the Biovia version of the CHARMm force field in DS 3.5. Each model was ‘relaxed’ with a brief (10 ps) molecular dynamics simulation at 300 K using the same backbone restraints.

### 4.8. Data Analysis

The results are presented as a percentage change in ATP EC_10_-activated currents (nA) after normalizing them with the response obtained with agonist alone. All the results are expressed as mean ± SEM. The data were obtained from oocytes from at least two different frogs. The n refers to the number of different oocytes tested. Significant differences were determined by non-parametric one-way ANOVA. Prism (GraphPAD Software, San Diego, CA, USA) was used to perform statistical analyses and curve fitting. The ATP concentration response data were fitted to a concentration-response curve using the logistic equation I/Imax = 100*[drug]^n^/([drug]^n^ + (EC_50_)^n^), where I/Imax is the percentage of the maximum obtainable response, EC_50_ is the concentration producing a half-maximal response, and n is the Hill coefficient (n*_H_*). Statistical significance was defined as *p* < 0.05.

## 5. Conclusions

The findings of the present study provide new insight into the role of residues in the TM1 segment in receptor activity and ethanol action in P2X4Rs. We demonstrate that position 49 contributes to the channel function by providing flexibility/stability of the upper portion of the alpha-helix during channel opening. These findings also suggest that R33 in the lower part of the TM1 segment is involved in ethanol sensitivity at lower, behaviorally relevant ethanol concentrations. Moreover, interactions between R33 in the TM1 segment of one subunit and D354 in the TM2 segment of the neighboring subunit may be important in affecting the channel transition from closed to open conformation and thus affect ion conduction, as well ethanol sensitivity. These results identify new residues that are important for ethanol action on P2X4Rs and, in combination with modeling studies, provide new information for the development of a pharmacophore for AUD drug discovery.

## Figures and Tables

**Table 1:** I_max_, EC_50_, and Hill slope values for ATP concentration-response curves of wild-type (WT) and TM1 segment mutant P2X4Rs. I_max_ represents the peak currents generated by 100 µM ATP. EC_50_ and Hill slope values were determined from ATP concentration-response curves. The data shown are mean ± SEM; individual *t*-test (non-parametric, Mann–Whitney), ^#^ *p* < 0.05, ^##^ *p* < 0.01 compared to WT P2X4Rs. One-way ANOVA, * *p* < 0.05, ** *p* < 0.01 compared to WT P2X4Rs.

Receptor	ATP			
	Imax, nA	EC50, μM	Hill Slope	N
WT P2X4	4215 ± 678	5.74 ± 0.45	1.48 ± 0.10	9
V49A	n.f.	n.d.	n.d.	7
F48A	8716 ± 1533 ^#^	3.53 ± 0.73 *	1.04 ± 0.09 *	8
V47A	2049 ± 319 ^#^	5.0 ± 0.76	1.27 ± 0.06	7
W46A	3230 ± 498	3.58 ± 0.57 *	1.02 ± 0.08 *	10
G45A	5994 ± 1100	4.65 ± 0.54	1.16 ± 0.05 *	10
I44A	4025 ± 687	5.68 ± 0.42	1.21 ± 0.05	11
V43A	6987 ± 1602	4.83 ± 0.59	1.34 ± 0.13	7
Y42A	1372 ± 119 ^##^	4.33 ± 0.7	1.11 ± 0.25	5
A41W	1667 ± 233 ^##^	4.62 ± 0.47	1.34 ± 0.07	12
L40A	1633 ± 282 ^##^	6.41 ± 0.73	1.50 ± 0.10	8
I39A	5544 ± 1510	5.87 ± 0.70	1.39 ± 0.07	7
L38A	5719 ± 1211	7.33 ± 1.05	1.32 ± 0.06	8
L37A	5688 ± 749	8.91 ± 0.84 **	1.20 ± 0.08	7
Q36A	1568 ± 129 ^#^	6.35 ± 1.81	1.33 ± 0.14	4
V35A	7079 ± 1491	6.35 ± 0.55	1.36 ± 0.10	6
A34W	4386 ± 608	6.50 ± 1.02	1.21 ± 0.06	11
R33A	3545 ± 748	7.04 ± 0.51	1.03 ± 0.02 *	5
N32A	2986 ± 397	5.40 ± 1.00	1.44 ± 0.08	7
M31A	3456 ± 375	7.07 ± 1.64	1.49 ± 0.23	7
L30A	7161 ± 1188	6.76 ± 0.84	1.37 ± 0.19	6
G29A	1175 ± 44 ^##^	6.43 ± 0.34	1.33 ± 0.10	8

n.f.—non-functional; n.d.—not determined.

**Table 2:** I_max_, EC_50_, and Hill slope values for ATP concentration-response curves of WT, position 49 and position 33 mutant P2X4Rs. I_max_ represents the peak currents generated by 100 µM ATP. The EC_50_ and Hill slope values were determined from ATP concentration-response curves. The data shown are mean ± SEM; one-way ANOVA, * *p* < 0.05 compared to WT P2X4Rs.

Receptor	ATP			
	Imax, nA	EC50, μM	Hill Slope	N
WT P2X4	5425 ± 1235	5.45 ± 0.4	1.47 ± 0.13	8
V49P	n.f.	n.d.	n.d.	5
V49G	2131 ± 292 *	9.60 ± 0.9 *	1.23 ± 0.10	6
V49L	2365 ± 277	10.60 ± 1.3*	1.54 ± 0.18	4
R33K	5428 ± 986	6.33 ± 0.93	1.35 ± 0.09	9
R33S	2492 ± 912	4.94 ± 1.09	1.35 ± 0.22	6
R33V	5947 ± 1396	3.67 ± 1.28	0.86 ± 0.08 *	6
R33C	5214 ± 560	3.96 ± 2.03	1.24 ± 0.36	7
R33F	4270 ± 791	4.68 ± 1.14	1.03 ± 0.03	7
R33L	2539 ± 418 *	5.06 ± 0.70	0.85 ± 0.12 *	9
R33Y	n.f.	n.d.	n.d.	11
R33Q	n.f.	n.d.	n.d.	3
R33E	n.f.	n.d.	n.d.	3

n.f.—non-functional; n.d.—not determined.

**Table 3:** I_max_, EC_50_, and Hill slope values for the ATP concentration-response curves of WT, position 33 and position 354 reciprocal mutants injected without and with the WT P2X4R. I_max_ represent peak currents generated by 100 µM ATP. EC_50_ and Hill slope values were determined from ATP concentration-response curves. The data shown are mean ± SEM.

Receptor	ATP			
	Imax, nA	EC50, μM	Hill Slope	N
WT P2X4	2886 ± 614	5.34 ± 0.11	1.11 ± 0.08	7
R33D	n.f.	n.d.	n.d.	5
D354R	n.f.	n.d.	n.d.	6
R33D-354R	n.f.	n.d.	n.d.	6
R33D:WT,1:1	763.3 ± 519	5.84 ± 0.11	1.45 ± 0.11	7
D354R:WT, 1:1	560 ± 195	7.24 ± 1.07	1.2 ± 0.95	5
R33D-D354R:WT, 1:1	540 ± 143	4.94 ± 1.06	1.2 ± 0.09	6

n.f.—non-functional; n.d.—not determined.

## Abbreviations

AUD	alcohol use disorder
ATP	adenosine triphosphate
EC	effective concentration
P2 × 4R	purinergic P2X4receptor
*p2rx4*	gene encoding P2 × 4R
TM	transmembrane
WT	wild-type
I_max_	maximum inducible current
IVM	ivermectin
